# High precision CA-ID-TIMS U-Pb zircon age for the “Dueling Dinosaurs” locality, with implications for regional correlation, basal age and duration of the Hell Creek Formation, Montana

**DOI:** 10.1371/journal.pone.0328861

**Published:** 2026-05-20

**Authors:** Eric M. Roberts, Marc S. Hendrix, Jahandar Ramezani, William C. Clyde, Pierre Zippi, Stuart Hodgson, Valerie Yuleridge, Lindsay E. Zanno

**Affiliations:** 1 Department of Geology and Geological Engineering, Colorado School of Mines, Golden, Colorado, United States of America; 2 Department of Geosciences, University of Montana, Missoula, Montana, United States of America; 3 Department of Earth, Atmospheric and Planetary Sciences, Massachusetts Institute of Technology, Cambridge, Massachusetts, United States of America; 4 Department of Earth Sciences, University of New Hampshire, Durham, New Hampshire, United States of America; 5 Biostratigraphy.com, LLC, Garland, Texas, United States of America; 6 Department of Earth Sciences, Southern Methodist University, Dallas, Texas, United States of America; 7 Earth and Environmental Sciences, College of Science and Engineering, James Cook University, Townsville, Queensland, Australia; 8 Department of Biological Sciences, North Carolina State University, Raleigh, North Carolina, United States of America; 9 North Carolina Museum of Natural Sciences, Raleigh, North Carolina, United States of America; 10 Department of Geology, Field Museum of Natural History, Chicago, Illinois, United States of America; 11 Paleontology, Natural History Museum of Utah, Salt Lake City, Utah, United States of America; 12 Vertebrate Paleontology, Sam Noble Museum, Norman, Oklahoma, United States of America; 13 Department of Earth Sciences, Stellenbosch University, Private Bag X1 Matieland, Stellenbosch, South Africa; 14 Evolutionary Studies Institute, University of Witwatersrand, Johannesburg, South Africa; New Mexico Museum of Natural History & Science, UNITED STATES OF AMERICA

## Abstract

Discovery of the “Dueling Dinosaurs” and other significant dinosaur localities from remote and isolated exposures of the Hell Creek Formation in central Montana highlight the complexity of establishing stratigraphic context and correlating Hell Creek Formation fossil localities located within and outside of the type area. Stratigraphic correlation is particularly problematic for the lower two-thirds of the Hell Creek Formation, which generally lacks reliable biostratigraphic or magnetostratigraphic zonation and has no dated ash beds. To address these enduring issues for one of the most significant Upper Cretaceous terrestrial fossil-bearing units in North America, detailed stratigraphic sections were established on the Murray Ranch and on McGinnis Butte in central Montana and correlated with other published Hell Creek Formation localities via magnetostratigraphy, biostratigraphy, and radioisotopic dating of ash beds. Results indicate that the K-Pg boundary is not exposed in the study area; however, high-precision U-Pb CA-TIMS zircon ages for two newly discovered ash beds (66.929 ± 0.020 Ma and 66.850 ± 0.026 Ma, 2σ internal uncertainties) that bracket the “Dueling Dinosaurs” quarry provide the first absolute ages for the lower portion of the Hell Creek Formation anywhere. Bayesian age-stratigraphic modelling places the “Dueling Dinosaurs” depositional age at 66.897 + 0.023/-0.028 Ma and suggests that the age of the base of the formation is ~ 67.102 + 0.710/-0.173 Ma (or older) in the study area. Comparison of stratigraphic architecture within the study area with published sections in the type area suggests that named sandstone marker horizons used for lithostratigraphic and sequence stratigraphic correlation in the type area have limited utility for regional correlation and need to be used with caution.

## Introduction

The Hell Creek Formation is one of Earth’s most iconic terrestrial rock records because it contains a diverse, abundant, and well-preserved vertebrate fauna (especially dinosaurs) and flora that existed immediately prior to the Cretaceous/Paleogene (K-Pg) boundary. Studied for over a century [1], the richly fossiliferous strata of the Hell Creek Formation remain a focus of investigation by researchers attempting to understand the tempo and mode of evolution and the causes, timing, and nature of extinction at the end of the Cretaceous [2–18]. Recent paleontological and geological investigations have focused on the stratigraphy of the uppermost Hell Creek Formation and the K-Pg boundary [19–21]. Sprain et al. [20–21] reported over 60 distinct tephra deposits over a 70 m interval spanning the uppermost Hell Creek Formation and overlying Fort Union Formation south of Fort Peck Reservoir close to the type area. This work has resulted in a high level of temporal and stratigraphic resolution for this interval based on biostratigraphy, magnetostratigraphy, chemostratigraphy and a series of high-precision radioisotopic ages of ash beds. Although the uppermost Hell Creek Formation is stratigraphically and temporally well-resolved, the lower two-thirds of the formation is not, and no radioisotopic ages have been reported. This lack of absolute age control in most of the formation is surprising because the entire formation is richly fossiliferous and is one of the premier sources of Upper Cretaceous terrestrial vertebrates and plant macrofossils in North America [6,10]. Moreover, age estimates for the base of the formation and total duration of the formation vary widely, and remain a point of confusion [19,22–26].

As greater attention is being devoted to establishing a high-resolution chronostratigraphic framework for the Campanian-Maastrichtian of the Western Interior Basin [20,21,27–35], the middle to lower portions of the Hell Creek Formation represent a critical stratigraphic interval in need of better documentation, particularly in central and southern Montana, Wyoming and South Dakota. Most stratigraphic studies of the formation have been focused on the Fort Peck Reservoir area north of Jordan, MT (lectostratotype area) and in portions of western-central North Dakota [6,21,36,37], with very few studies outside of these two areas.

In recent years, exposures of the Hell Creek Formation southwest of Jordan, Montana have drawn attention for the preservation and excavation of significant dinosaur discoveries made on private land. Unfortunately, many dinosaur specimens recovered from private land lack the same level of stratigraphic and taphonomic context as those excavated in the more well-studied exposures along Fort Peck Reservoir and in North Dakota, thereby limiting their scientific value. Perhaps the most publicized fossil discovery of this nature is the “Dueling Dinosaurs” [38], a unique fossil preserving nearly complete, entwined skeletons of *Nanotyrannus lancensis* (NCSM 40000) [39] and a *Triceratops* (NCSM 40001) hypothesized in public forums to have died locked in combat.

**Fig 1:**
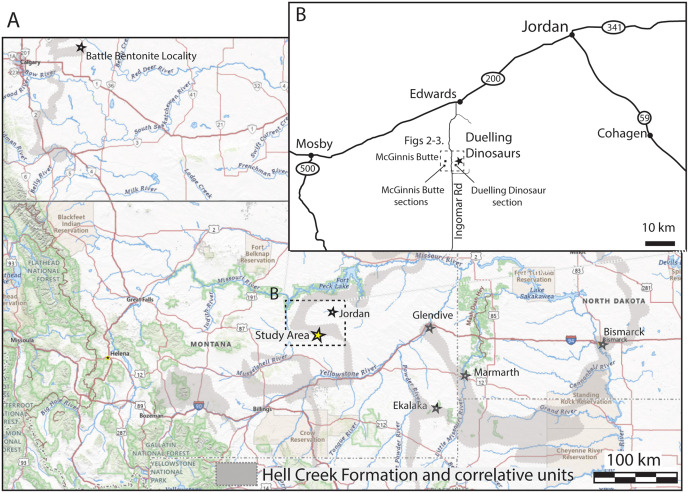
Study area. (A) Map showing the location of the Study Area (yellow star) and other important Hell Creek Formation and correlative formation locations (white stars). Gray shading shows the approximate distribution of the Hell Creek Formation and correlative strata. Data from [6,14,25] and map from USGS National Map Viewer (https://apps.nationalmap.gov/viewer/). **(B)** Detailed location map of the “Dueling Dinosaurs” site and key measured sections within the study area. (Map modified from USGS National Map Viewer; https://apps.nationalmap.gov/viewer/).

NCSM 40000 and 40001 were collected over two decades ago as part of commercial excavation. When gifted to the NCSM limited details were available as to the specimens’ stratigraphic and sedimentological context. As a first step towards contextualizing this important locality and encouraging similar investigations of other sites with limited geological context, we returned to the original excavation site of the “Dueling Dinosaurs” (NCSM 40000 and 40001) and nearby McGinnis Butte with permission of the landowners and original collectors to establish a robust Hell Creek Formation reference section calibrated by high precision chronostratigraphy (Fig 1). Key goals of this study were 1) to identify datable volcanic ash beds or bentonites from the lower two thirds of the Hell Creek Formation, along with supporting magnetostratigraphic and palynologic data to better resolve the age of the fossils; 2) to establish constraints on the total duration of the Hell Creek Formation in this area (and regionally); and 3) to better document the stratigraphic relations between the Hell Creek Formation at this locality and correlative sections and units in the US and Canada.

## Geologic background

The Hell Creek Formation is an exceptionally well-exposed and continuous Upper Cretaceous (Maastrichtian) formation that crops out over parts of central to eastern Montana as well as western North and South Dakota (Fig 1). Initial study of the Hell Creek Formation was undertaken by paleontologist Barnum Brown between 1902 and 1909. Brown [1] identified a series of sandstone and mudstone units above the underlying Fox Hills Formation and below the overlying lignitic beds of the Fort Union Formation. Later work by Cobban and Reeside [40] elevated these beds to the formation level. More recent studies of the Hell Creek Formation have tended to divide the formation into the informal lower, middle, and upper Hell Creek designations [10,11,26], although Frye [41] and others have suggested as many as seven members. Despite an apparent convergence towards a regional stratigraphic understanding of the Hell Creek Formation in more recent works, the formation is infamous for the complexity of its lateral facies relationships, with many stratigraphic units pinching out laterally and interfingering with facies that represent different sub-environments [7,42]. Indeed, many workers have noted a paucity of true marker horizons in the formation that prevented regional correlation within the Hell Creek Formation except when either the upper or lower formation boundary could be observed [6,11,26]. Since Brown [1] did not formally define a stratotype section in his original description of the formation, the subsequent designation of a lectostratotype section at Flagg Butte (along a tributary of Hell Creek) by Hartman et al. [11] has alleviated some of these issues, particularly in and around the Fort Peck Lake region. However, it is unclear how comparable the Hell Creek Formation lectostratotype is outside of the Fort Peck region, and how well it applies to other fossiliferous sites that crop out tens or hundreds of kilometers away.

The Hell Creek Formation is also laterally equivalent (at least partially) to a number of other formations in the US and Canada, and the precise stratigraphic relationships remain undocumented in most cases, with limited information on the paleogeographic and paleoenvironmental variations between contemporary units. Correlative units include the Lance Formation in Wyoming and NE Colorado [6,43], the Frenchman and Scollard formations in Saskatchewan and Alberta, Canada [25], the Denver Formation in eastern Colorado [44], the North Horn Formation in Utah [45], the Ojo Alamo Formation and McRae Group in New Mexico [46–48] and the Javelina Formation in Texas [49].

The Hell Creek Formation is characterized by a strongly heterogeneous series of interbedded sandstone and mudstone/siltstone units that are notably fossiliferous [1,11]. The contact with the overlying Fort Union Formation forms the Cretaceous/Paleogene (K-Pg) Boundary, an event associated with the extinction of all non-avian dinosaurs, as well as many other floral and faunal species [2,13,42,49–55]. Several marine tongues have been identified locally within the Hell Creek Formation, including the Breien Member and Cantapeta Tongues in North Dakota [7]. Otherwise, the Hell Creek Formation depositional environment has been interpreted as an extensive floodplain with meandering river systems and ephemeral backwater deposits [56]. The sandstone units are usually interpreted as large, extensive river systems [57] or channel complexes [26].

A significant amount of geochronological work has been conducted on the Hell Creek Formation, mostly focusing on the Hell Creek – Fort Union formational contact, due to its association with the K-Pg Boundary [42,58]. The placement of the upper contact with the end-Cretaceous impact event (at least on a regional scale) constrains the upper age of the formation to ~66.052 ± 0.008/0.043 Ma [20,21,53], based on radiometric ages of numerous tephra-containing lignite beds common above and below the contact [20–21]. The age of the upper Hell Creek is also relatively constrained, thanks to an ash bed from an isolated coal seam (the Null Coal), which was dated via ^40^Ar/^39^Ar geochronology by Sprain et al. [20], yielding an age of 66.289 ± 0.051 Ma. However, no radioisotopic age determination has been conducted on strata below this level. As a result, age estimates for the base of the Hell Creek Formation, primarily based on magnetostratigraphy, are inconsistent and range from 68.369 to 68.196 Ma [59], 67.47 to 67.5 Ma [37], 66.87 to 66.71 Ma [22]. Uncertainty surrounding sediment accumulation rates and the total duration of the Hell Creek Formation has, in part, been attributed to the high degree of erosional scouring noted at the base of many of the fluvial sandstone units in the formation and apparent lateral variability between locations [26]. Collectively, stratigraphic and geochronologic uncertainties have led to differences in the estimated duration of deposition for the Hell Creek Formation that vary by >200%, ranging from 1.86 Myr [9], 1.36 Myr [22], 1.16 Myr [25], to 0.9 Myr [26]. This issue is not necessarily related to inaccurate dating in earlier studies, but rather a combination of likely issues, including the presumed time transgressive nature of the contact with the underlying Fox Hills Formation, and/or localized erosion or nondeposition at the contact in places.

## Methods

The focus of this investigation was to establish reference stratigraphic sections both at the “Dueling Dinosaurs” locality and nearby locations to build a well-constrained composite section in the region. Fieldwork was conducted at the “Dueling Dinosaurs” site, as well as various other locations on the Murray Ranch and at the nearby McGinnis Butte section between 2018 and 2025. Three stratigraphic sections were hand-trenched and measured with a Jacob Staff. A full section through the study area was measured in two parts and called the lower and upper McGinnis Butte Sections (MB), and ~2 km to the east, a partial section was measured through the “Dueling Dinosaurs” Quarry, termed the Dueling Dinosaurs (DD) section. This work was coupled with careful collection of palynological and paleomagnetic samples, as well as sampling of volcanic tuff/bentonite layers on the Murray Ranch for U-Pb geochronology.

### Palynology

Seventeen samples were collected for palynological investigation from hand-trenched sections (~20–45 cm deep) to avoid surficial contamination and to obtain relatively unweathered samples. Sampling was focused on the upper parts of both the MB and DD sections to help locate the contact with the Fort Union Formation and the K-Pg boundary. After lithological examination and description, the samples were processed for palynology preparation. The samples were washed to remove surficial contaminants. Carbonate minerals were dissolved using HCl, and silicate minerals removed using HF. After a wash with hot HCl, heavy liquid separation was performed with ZnBr_2_. The organic residue was washed with cold Schultz’s solution, followed by a wash with ammonium hydroxide. The residues were sieved through a 7-μm mesh screen to remove small particles that would be unidentifiable in transmitted light microscopy. Organic residues were mounted on a coverslip with polyvinyl alcohol and fixed to a microscope slide with polyester resin. Slides were examined with phase contrast and differential interference contrast illumination using oil immersion at a minimum of 500X with a research-grade Zeiss Axio Imager microscope. When possible, palynomorph occurrence data were collected until the total count reached 100 specimens for relative abundance data, after which, the remaining area of the slide was scanned for rare taxa that may have stratigraphic significance. Rare taxa were added to the count data. Key specimens were photographed, and original slides stored at Colorado School of Mines.

### Magnetostratigraphy

Oriented paleomagnetic hand samples were collected from indurated fine-grained units by removing cover to expose fresh, relatively unweathered surfaces. No tilt correction was required since the beds are essentially horizontally oriented in the study area. Due to the poorly cemented, friable nature of many of the samples, we were not always able to collect replicate samples, and in some cases returned to the site to collect additional samples. Sampling strategy for paleomagnetic sampling was conducted following the acquisition of palynological analysis and radioisotopic ages of the two bentonite horizons, with the specific goal of locating the C30N/C29R and C29R/C29N reversals to establish stratigraphic control for the top of the section in this area and potentially identify the bounds of Chron C29R that contains the K-Pg boundary. Stratigraphic spacing between sample sites varied but was typically between <1 m to 5 m through the primary interval of interest due to variability in the availability of appropriate lithologies. Five stratigraphic levels (= “sites”) were sampled through the upper DD section, ranging from the DD quarry to the top of the local section, and ten stratigraphic levels were sampled through the MB Section.

Samples from each location were cut into 8 cm^3^ cubes, retaining the oriented surface as one cube face, and analyzed at the University of New Hampshire Paleomagnetism Laboratory. Samples were measured using a 2G Enterprises superconducting quantum interference device (SQUID) cryogenic magnetometer shielded from the background magnetic field, and step-wise demagnetized using a combination of an ASC Scientific Model TD-48SC thermal demagnetizer and AF Molspin tumbling alternating field (AF) demagnetizer. A mixed thermal and AF demagnetization protocol is consistent with the many previous paleomagnetic studies of the Hell Creek Formation, which suggest titanomagnetite as the dominant NRM carrier [3,4,19,21,22,37] with intermediate titanohematite and goethite as potentially important ancillary magnetic carrier phases [60].

All sample data were analyzed using the PuffinPlot paleomagnetic data program [61]. Samples with three or more sequential steps exhibiting linear or quasi-linear decay to the origin were characterized using principal component analysis (PCA; [62]). Only those samples with a maximum angular deviation of ≤ 22° were included. A Fisher mean [63] was calculated for samples displaying an initial decay followed by strong clustering of vector endpoints and no further decay. Some samples with overlapping unblocking spectra display a demagnetization path best characterized by a great circle. Some samples exhibited unstable demagnetization behaviors and were excluded from further analysis. The mean virtual geomagnetic pole latitude for each site was calculated and used to determine its polarity. Samples are stored at University of New Hampshire.

### U-Pb geochronology by the CA-ID-TIMS method

**Fig 2:**
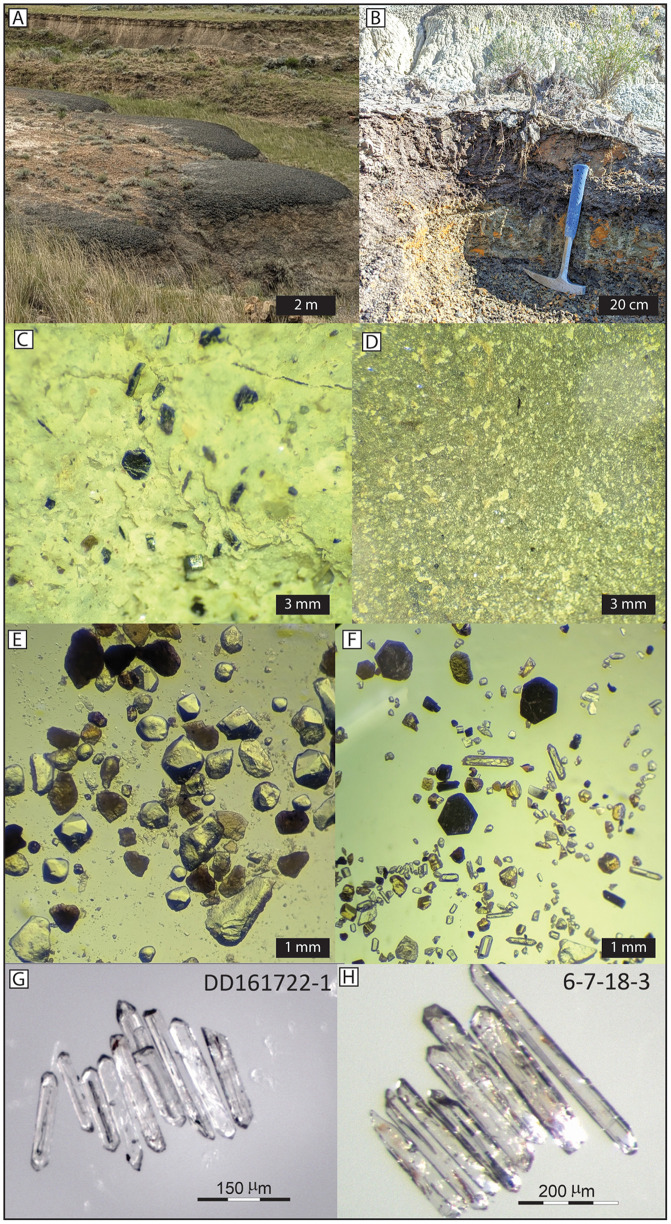
Photographs of the Dueling and Ingomar Bentonites. (A) Field view of the Ingomar Bentonite after a rainstorm, resulting in a swollen, dark, popcorn texture that is distinctive from other sedimentary facies. (B) Excavated view of the Dueling Bentonite, revealing a distinctive pistachio green color when freshly exposed. The ~ 20 cm-thick Dueling bentonite is located several meters below the “Dueling Dinosaurs” fossil site (NCSM 40000 and NCSM 40001) and was deposited into a carbonaceous shale, making it stand out and easier to see top and bottom contacts and to tell that it has not been reworked. (C, D) Close-up view of fresh bentonitic claystone with abundant biotite phenocrysts in the Dueling and Ingomar bentonites, respectively. The fresh, euhedral biotites and lack of detrital sand indicate that both are primary ash fall deposits. (E) Stereomicroscope image of the light mineral fraction in the Dueling Bentonite is composed of broken, angular quartz and sanidine phenocrysts as well as euhedral biotite and bipyramidal quartz crystals. In addition, the key characteristic of both bentonites that distinguishes them from reworked bentonite is their lack of rounded, detrital grains. (F) Stereomicroscope image of the heavy mineral separates from the Dueling Bentonite shows fresh euhedral biotite, zircon, apatite, and titanite. Note the presence of multiple long, acicular zircon phenocrysts with large melt inclusions indicative of eruption-phase zircon crystals. (G and H). Stereomicroscope images of selected zircons for U-Pb analysis from the Ingomar (G) and Dueling (H) bentonites.

Identification, collection, and tracing of two distinct bentonite beds, interpreted herein as altered pyroclastic ash-fall deposits, were carefully conducted to identify locations where the beds, which pinch in and out, were each purest. The two beds, termed the Dueling and Ingomar bentonites, are ~ 4–5 m apart stratigraphically and can both be traced through the outcrop area; however, the two beds were sampled in separate locations ~2 km apart from each other (Fig 2). This was done by digging large pits and excavating down onto the bentonite surface to collect fresh, uncontaminated samples (4–5 kg of material) from several mm above the basal surface of the bentonite (to avoid contamination with the underlying unit). Samples were processed in the lab by soaking in water for 1–2 days, followed by liquefaction using a blender and gradual clay disaggregation via a sonic dismembrator [64]. After reducing the sample to sand and silt-sized grains (mostly volcanic phenocrysts), paramagnetic minerals were removed using a Franz magnetic separator, followed by a high-density liquid separation using methylene iodide to produce the heavy mineral separate. Final zircon selection was carried out by hand picking under a binocular microscope, in which preference was given to sharply faceted, acicular zircon that contained elongate glass (melt) inclusions parallel to their long axis. Ramezani et al. [31] demonstrated in other Cretaceous Western Interior bentonite samples that these grains tend to yield the youngest eruption phase ages. Remaining mineral separates are stored at Colorado School of Mines.

Zircon U-Pb analyses by the chemical abrasion thermal ionization mass spectrometry (CA-ID-TIMS) method were carried out at the Massachusetts Institute of Technology Isotope Laboratory, following the detailed procedures described in Ramezani et al. [31]. Zircons selected for U-Pb dating were pre-treated by a chemical abrasion technique modified after Mattinson [65]{Mattinson, 2005 #8}, which involved thermal annealing in a 900°C furnace for 60 hours, followed by partial dissolution (leaching) in concentrated hydrofluoric acid (HF) to mitigate the effects of Pb-loss in zircon that result in anomalously young dates [31]. For leaching, annealed zircons were loaded with ~75 µl of 29 M HF into 200 µl FEP Teflon® microcapsules, placed within a high-pressure Parr® vessel, and left in a 210°C oven for 12–13 hours. This is considered a rather aggressive leach schedule but proven necessary as a remedy for persistent Pb loss in certain zircons (Widmann et al., 2019). The leached grains were transferred into 3 ml Savillex® FEP beakers and fluxed in successive steps in a dilute HNO_3_ solution and in 6N HCl over a hot plate (1 hour per step), with each step followed by agitation in an ultrasonic bath (1 hour) and rinsing with several milliliters of ultra-pure water to remove the leachates. Thoroughly rinsed zircon grains were loaded back into their microcapsules, spiked with the EARTHTIME ET2535 mixed ^202^Pb-^205^Pb-^233^U-^235^U isotopic tracer [66,67], and dissolved completely in 29M HF at 210°C for 48 hours.

Dissolved Pb and U were chemically separated using a miniaturized HCl-based ion-exchange chemical procedure modified after Krogh [68], using 50 µl columns of 1x8 anion-exchange resin. Purified Pb and U were loaded with a silica gel – H_3_PO_4_ emitter solution [69] onto single, degassed Re filaments, and their isotopic ratios were measured on the Isotopx X62 multi-collector thermal ionization mass spectrometer equipped with a Daly photomultiplier ion counting system at MIT. Pb isotopic measurements were made on monoatomic Pb ions in a peak-hopping mode on the ion counter, whereas U isotopes were measured as UO_2_^+^ in a static mode on three Faraday detectors simultaneously.

Five zircons each were analyzed from the two bentonite beds in the Hell Creek Study Area. Data reduction, as well as calculation of U-Pb dates and propagation of uncertainties, were accomplished using the Tripoli and ET_Redux applications [70,71]. Measured isotopic ratios were corrected for mass-dependent isotope fractionation in the mass spectrometer using the tracer ^202^Pb/^205^Pb and ^233^U/^235^U isotopic ratios, as well as for U and Pb contributions from the spike and laboratory blanks. Common Pb in the analyses averaged 0.37 pg, all of which was attributed to laboratory blank, and its isotopic composition was determined from long-term measurements of the total procedural Pb blank in the lab (see Table DR1 footnotes). The radiogenic ^206^Pb concentrations were also corrected for initial ^230^Th disequilibrium in magma using a magma initial Th/U model ratio of 2.8 ± 1.0 (2σ). This range of Th/U ratios encompasses all likely compositions of the magma source of an intermediate to felsic tuff [e.g., 72]. Pb isotopic ratios were corrected for isobaric interferences from Tl and BaPO_2_ on mass 205 by monitoring masses 203 and 201, respectively, and using natural isotopic abundances of ^138^Ba and ^205^Tl. Measured U isotopic ratios were also corrected for isobaric interference of ^233^U^18^O^16^O with ^235^U^16^O^16^O using an ^18^O/^16^O ratio of 0.00205 ± 0.00004 (2σ), which has been determined from long-term measurements of 272/270 mass ratio from large U loads. The present-day natural U isotopic composition of 137.818 ± 0.044 (2σ) was used in data reduction following [73].

In general, ^206^Pb/^238^U dates are considered the most precise and accurate in high-precision U-Pb geochronology as they are independent of suspected inaccuracy of the ^235^U decay constant [74,75], which potentially biases the ^207^Pb/^235^U or ^207^Pb/^206^Pb dates. Sample ages are derived from the weighted mean ^206^Pb/^238^U date of the five analyzed zircons in each sample, which form statistically coherent clusters. No analyses were rejected. Uncertainties in calculated ^206^Pb/^238^U dates are reported at 95% confidence level and in the ± *X*/*Y*/*Z* Ma format, where *X* is the internal (analytical) uncertainty in the absence of all external errors, *Y* incorporates *X* and the U-Pb tracer calibration errors, and *Z* includes the latter as well as the decay constant errors of Jaffey et al. [76]. U-Pb bentonite ages presented here are discussed using the analytical uncertainty (X). When comparing these ages with data collected using different tracer solutions, analytical methods, or isotopic chronometers, consideration must be given to systematic contributions to uncertainty (i.e., Y and Z factors).

### Age-stratigraphic modelling

To construct a robust chronostratigraphic framework for the Hell Creek Formation at the “Dueling Dinosaurs” study area, we first established a composite stratigraphic section based on correlation of the DD and MB sections. To correlate the DD and MB sections, we walked out the Dueling and Ingomar bentonite beds between the sections, and we walked out the top contact of a prominent sandstone unit to correlate the Lower and Upper MB sections. We then employed a Bayesian age-stratigraphic model of the composite section generated using the Bchron software package [77,78]. The model utilizes the weighted mean dates of the two radioisotopically-dated bentonites and a recalculated age for the C30n/C29r boundary based on high precision geochronology and magnetostratigraphy from the Denver Basin [27,79] modelled using the same Bayesian age-stratigraphic approach employed herein (66.48 + 0.55/-0.17 Ma; larger of these asymmetric uncertainties was used in the DD-MB model) (see data Tables S1-S6 in S1 File in Supplementary Materials). These input ages along with their relative stratigraphic positions were used to interpolate the age of the “Dueling Dinosaurs” site and the base of the formation. The underlying Markov chain Monte Carlo rejection algorithm of Bchron considers possible changes in the sediment accumulation rate and results in more objective stratigraphic age uncertainties than the conventional linear extrapolation or spline-fit methods. The Bchron age model is shown with its median (solid) line and 95% confidence interval (shaded band). Code scripts, input data, and numerical model outputs for the DD-MB model and Denver Basin (Kiowa Core) after [27,34] are included in Supplementary Materials (Tables S1-S6 in S1 File).

## Results

### Lithostratigraphy

**Fig 3:**
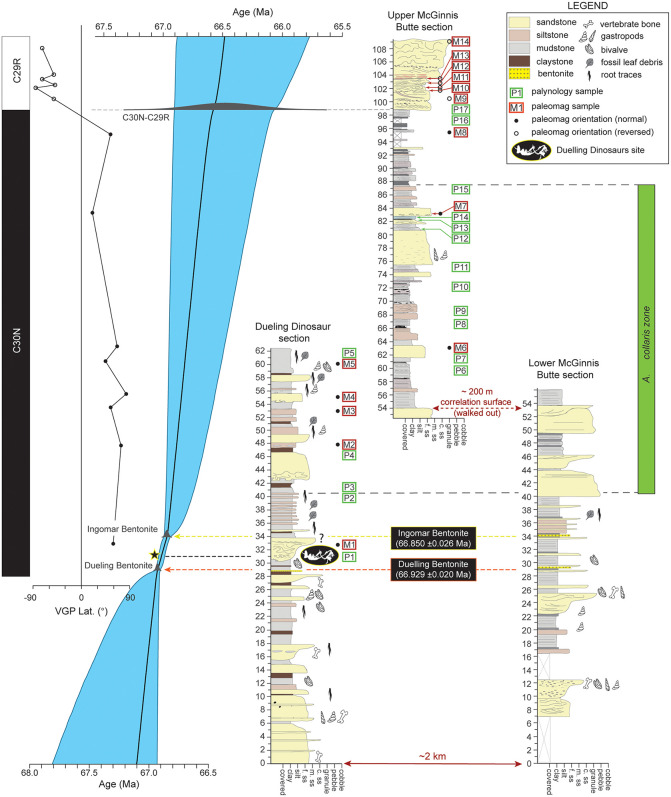
Correlated measured sections through the study area with accompanying chronostratigraphy and palynostratigraphy, with Bayesian age-stratigraphic model on the left. Note that pollen and paleomagnetic site locations are shown by green and red boxes, respectively.

Three stratigraphic sections were measured (lower and upper MB sections and DD Section; Figs 1 and 2) to establish a continuous composite stratigraphic framework through the study area. Sections were precisely correlated by tracing bentonite marker horizons between sections and walking out a continuous sandstone bench between the lower and upper MB sections (Figs 2 and 3). Although the underlying Fox Hills Formation was clearly identified in isolated locations throughout the study area, we are not entirely certain whether the proposed contact observed at the base of the Dueling Dinosaurs Section and lower McGinnis Butte section represents the actual contact between Fox Hills and the Base of the Hell Creek Formation. The proposed top of the Fox Hills Formation was observed at both sections and is marked by a major, multistory sandstone bench with persistent Fe-oxide staining and concretion development. In nearby locations (within 0.5 km) where the Fox Hills Formation is better exposed, similar Fe-oxide staining and concretions are present. However, the contact between the Fox Hills and Hell Creek formations has been described as highly variable from location to location [e.g., 26]. Complicating things further, we saw no evidence of the ‘toothpaste’ marker horizon in the study area or the Colgate Sandstone [80] in the area, which sometimes defines the Hell Creek-Fox Hills contact. Hence, we prefer to interpret the Fe-Oxide-stained concretionary sandstone bench as the contact, given that we see mostly covered interval below this level in the study area, with only isolated outcrops of Fox Hill Formation lower in section. Given this uncertainty, we must consider the composite thickness of 109 m measured in the study area to be a minimum thickness for the Hell Creek Formation in the region (Fig 3).

### Palynology

**Fig 4:**
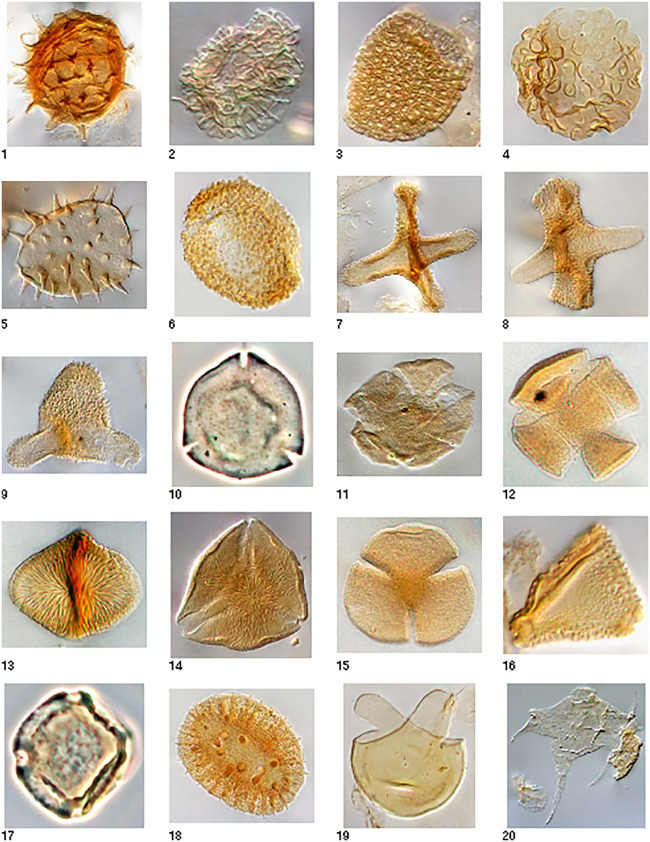
Photographs of diagnostic spores and palynomorphs documented in the Hell Creek Formation in the DD and MB sections. 1: *Ghoshispora bella*, 75 μm, P11 (9/94), L Maastrichtian-E Paleocene; 2: *Liliacidites altimurus* 26 μm, P3 (9.5/89), L Maastrichtian; 3: *Liliacidites complexus*, 40 μm, P5 (4/94), L Campanian-L Maastrichtian; 4: *Marsypiletes cretacea,* 44 μm, P1, Maastrichtian; 5: *Nuphar/Nypa/ Spinizonocolpites* sp., 45 μm, P3 (10/92.5), Latest Maastrichtian-Recent; 6: *Racemonocolpites formosus*, 52 μm, P9 (9/88), Latest Maastrichtian, except for one Latest Maastrichtian to Earliest Paleocene in Alberta; 7: *Aquilapollenites collaris*, 63μm, P11 (9/97), L Maastrichtian; 8: *Aquilapollenites conatus*, 54 μm, P3 (7/82.5), L Maastrichtian; 9: *Aquilapollenites delicatus*, 44 μm, 61822−4 10/98.5, L Maastrichtian; 10: *Kurtzipites circularis,* 23 μm, P9 (9/85), Maastrichtian-mid Paleocene; 11: *Leptopecopites pocockii*, 32 μm, P15 (12.5/76), Latest Maastrichtian; 12: *Scabrastephanocolpites lepidus*, p. 985, 25 μm, P9 (10/102), Campanian-Maastrichtian; 13: *Striatellipollis striatellus,* 28 μm, P8 (10/94), mid-L Maastrichtian; 14: *Styxpollenites calamitas*, 38 μm, P3 (9.5/89.5), Latest Maastrichtian; 15: *Tricolpites microreticulatus*, 24 μm, P7 (10/91), mid-L Maastrichtian; 16: *Tschudypollis retusus*, 23 μm, P11 (10/93.5), mid-Coniacian-L Maastrichtian; 17: *Ulmipollenites krempii*, 25 μm, P7 (10/92), L Maastrichtian-Recent; 18: *Wodehouseia spinata*, 50 μm, P1 (9/98.5), Latest Maastrichtian-Earliest Paleocene; 19: *Sigmopollis psilatus*, 29 μm, P11 (9/92), Maastrichtian-Recent; 20: *Nyktericysta davisii*, 95 μm, P2 (6.5/92), reworked from Middle-Late Albian marine strata.

Palynomorph recovery and preservation ranged from marginal to excellent in the 13 samples analyzed, and a total of 107 individual species were identified, with all species indicating a terrestrial or freshwater origin associated with fluvial, paludal, and lacustrine sub-environments (Table 1; Fig 4). Pollen diversity ranges from sample to sample. Some samples contain a low diversity palynoflora dominated by swamp fern spores and aquatic algae, suggesting local source derivation and slow-moving or ponded water (e.g., samples P6, P7, and P8). Other samples contain a moderate or high diversity palynoflora that includes reworked mid-Cretaceous spores and dinocysts, suggesting closer connection to a fluvial channel system (e.g., samples P9 and P13). A few samples were dominated by swamp fern spores, suggesting floodplain deposition with reworked pollen indicating contributions from overbank flooding (e.g., sample P11). Except for some reworked middle Cretaceous marine dinoflagellates, no marine palynomorphs were encountered.

**Table 1:** Palynological summary.

Sample #	Strat. Section	Strat. Level (m)	Sample age	Sample age confidence	First Appearance (FA) species	FA species age	Last Appearance (LA)	LA species age
P1 (21-DD-200)	DD	30.6	Latest Maastrichtian	high	*Ephedripites multipartitus*	Late Maastrichtian	*Aquilapollenites quadrilobus*	Latest Maastrichtian
					*Marsypiletes cretacea*	Latest Maastrichtian	*Ephedripites multipartitus*	Late Maastrichtian
					*Pandaniidites typicus*	Latest Maastrichtian	*Leptopecopites pocockii*	Late Maastrichtian
					*Ulmipollenites krempi*	Late Maastrichtian	*Marsypiletes cretacea*	Latest Maastrichtian
					*Ulmoideipites tricostatus*	Late Maastrichtian	*Tricolpites (Gunnera) microreticulatus*	Late Maastrichtian
					*Wodehouseia spinata*	Latest Maastrichtian		
					*Ghoshispora bella*	Late Maastrichtian		
					*Triplanosporites sinuosus*	Late Maastrichtian		
P2 (21-DD-203)	DD	40.1	Late Maastr-Paleocene	high	*Ulmipollenites krempi*	Late Maastrichtian	*Ulmipollenites krempi*	Paleocene
					*Triplanosporites sinuosus*	Late Maastrichtian	*Cyathidites diaphana*	Paleocene
							*Triplanosporites sinuosus*	Paleocene
P3 (61822−4)	DD	42.1	Latest Maastrichtian	high	*Ghoshispora bella*	Late Maastrichtian	*Osmundacidites?*	Late Maastrichtian
					*Osmundacidites?*	Late Maastrichtian	*Aquilapollenites collaris*	Latest Maastrichtian
					*Triplanosporites sinuosus*	Late Maastrichtian	*Aquilapollenites conatus*	Latest Maastrichtian
					*Aquilapollenites collaris*	Latest Maastrichtian	*Aquilapollenites delicatus*	Late Maastrichtian
					*Erdtmanipollis cretaceus*	Late Maastrichtian	*Aquilapollenites quadrilobus*	Latest Maastrichtian
					*Liliacidites altimurus*	Latest Maastrichtian	*Cranwellia rumseyensis*	Late Maastrichtian
					*Styxpollenites calamitas*	Late Maastrichtian	*Liliacidites altimurus*	Latest Maastrichtian
					*Ulmipollenites krempii* 4-pore	Late Maastrichtian	*Liliacidites complexus*	Latest Maastrichtian
					*Ulmipollenites krempii* 3-pore	Late Maastrichtian	*Striatellipollis striatellus*	Late Maastrichtian
					*Wodehouseia spinata*	Latest Maastrichtian	*Styxpollenites calamitas*	Latest Maastrichtian
P4 (61822−8)	DD	46.8	Latest Maastrichtian	high	*Osmundacidites?*	Late Maastrichtian	*Osmundacidites?*	Late Maastrichtian
					*Triplanosporites sinuosus*	Late Maastrichtian	*Aquilapollenites collaris*	Latest Maastrichtian
					*Aquilapollenites collaris*	Latest Maastrichtian		
					*Erdtmanipollis cretaceus*	Late Maastrichtian		
					*Pandaniidites typicus*	Latest Maastrichtian		
					*Ulmipollenites krempii* 3-pore	Late Maastrichtian		
P5 (61822−12)	DD	61.8	Latest Maastrichtian	high	*Liliacidites altimurus*	Latest Maastrichtian	*Liliacidites altimurus*	Latest Maastrichtian
					*Pandaniidites typicus*	Latest Maastrichtian	*Liliacidites complexus*	Latest Maastrichtian
					*Wodehouseia spinata*	Latest Maastrichtian	*Aquilapollenites quadrilobus*	Latest Maastrichtian
P6 (21-MB-35)	MB	51.2	Latest Maastrichtian	high	*Wodehouseia spinata*	Latest Maastrichtian	*Aquilapollenites delicatus*	
					*Ghoshispora bella*	Late Maastrichtian		
					*Triplanosporites sinuosus*	Late Maastrichtian		
P7 (21-MB-03)	MB	53.3	Latest Maastrichtian	high	*Aquilapollenites collaris*	Latest Maastrichtian	*Aquilapollenites collaris*	Latest Maastrichtian
					*Pandaniidites typicus*	Latest Maastrichtian	*Aquilapollenites quadrilobus*	Latest Maastrichtian
					*Ulmipollenites krempi*	Late Maastrichtian	*Tricolpites (Gunnera) microreticulatus*	
					*Triplanosporites sinuosus*	Late Maastrichtian		
P8 (21-MB-10)	MB	58.7	Late Maastrichtian	high	*Ephedripites multipartitus*	Late Maastrichtian	*Ephedripites multipartitus*	Late Maastrichtian
					*Ulmipollenites krempi*	Late Maastrichtian	*Striatellipollis striatellus*	Late Maastrichtian
					*Triplanosporites sinuosus*	Late Maastrichtian		
P9 (21-MB-13)	MB	60.85	Latest Maastrichtian	high	*Cheno-Am*	Late Maastrichtian	*Aquilapollenites quadrilobus*	Latest Maastrichtian
					*Ephedripites multipartitus*	Late Maastrichtian	*Ephedripites multipartitus*	Late Maastrichtian
					*Erdtmanipollis cretaceus*	Late Maastrichtian	*Tricolpites (Gunnera) microreticulatus*	Latest Maastrichtian
					*Kurtzipites circularis/simplex*	Late Maastrichtian		
					*Pandaniidites typicus*	Latest Maastrichtian		
					*Racemonocolpites formosus*	Latest Maastrichtian		
					*Ulmipollenites krempi*	Latest Maastrichtian		
P10 (21-MB-19)	MB	64.8	Latest Maastrichtian	high	*Aquilapollenites collaris*	Latest Maastrichtian	*Aquilapollenites collaris*	Latest Maastrichtian
					*Erdtmanipollis cretaceus*	Late Maastrichtian	*Aquilapollenites delicatus*	Late Maastrichtian
					*Gabonisporis cristatus*	Late Maastrichtian	*Aquilapollenites quadrilobus*	Latest Maastrichtian
					*Ulmipollenites krempi*	Late Maastrichtian	*Leptopecopites pocockii*	Late Maastrichtian
					*Ghoshispora bella*	Late Maastrichtian		
					*Triplanosporites sinuosus*	Late Maastrichtian		
P11 (21-MB-26)	MB	67.7	Latest Maastrichtian	high	*Alnipollenites trina*	Late Maastrichtian	*Aquilapollenites collaris*	Latest Maastrichtian
					*Aquilapollenites collaris*	Latest Maastrichtian	*Aquilapollenites delicatus*	Late Maastrichtian
					*Liliacidites altimurus*	Latest Maastrichtian	*Liliacidites altimurus*	Latest Maastrichtian
					*Pseudoaquilapollenites conatus*	Latest Maastrichtian	*Pseudoaquilapollenites conatus*	Latest Maastrichtian
					*Ulmipollenites krempi*	Late Maastrichtian	*Tricolpites (Gunnera) microreticulatus*	Late Maastrichtian
					*Ghoshispora bella*	Late Maastrichtian	*Tschudypollis (Proteacidites) retusus*	Late Maastrichtian
					*Toroisporis major*	Late Maastrichtian	*Aquilapollenites quadrilobus*	Latest Maastrichtian
					*Triplanosporites sinuosus*	Late Maastrichtian		
P12 (21-MB-30)	MB	73.7	Late Maastrichtian	high	*Toroisporis major*	Late Maastrichtian	*Tricolpites (Gunnera) microreticulatus*	Late Maastrichtian
					*Triplanosporites sinuosus*	Late Maastrichtian		
P13 (21-MB-31)	MB	74.2	Latest Maastrichtian	high	*Alnipollenites trina*	Late Maastrichtian	*Aquilapollenites attenuatus*	Late Maastrichtian
					*Aquilapollenites collaris*	Latest Maastrichtian	*Aquilapollenites collaris*	Latest Maastrichtian
					*Ephedripites multipartitus*	Late Maastrichtian	*Ephedripites multipartitus*	Late Maastrichtian
					*Erdtmanipollis cretaceus*	Late Maastrichtian	*Liliacidites complexus*	Latest Maastrichtian
					*Kurtzipites circularis/simplex*	Late Maastrichtian	*Pseudoaquilapollenites conatus*	Latest Maastrichtian
					*Pandaniidites typicus*	Latest Maastrichtian	*Tricolpites (Gunnera) microreticulatus*	Late Maastrichtian
					*Pseudoaquilapollenites conatus*	Latest Maastrichtian		
					*Ulmipollenites krempi*	Late Maastrichtian		
					*Ulmoideipites tricostatus*	Late Maastrichtian		
					*Triplanosporites sinuosus*	Late Maastrichtian		
P14 (21-MB-33)	MB	75.1	Latest Maastrichtian	high	*Aquilapollenites collaris*	Latest Maastrichtian	*Aquilapollenites collaris*	Latest Maastrichtian
					*Cheno-Am*	Late Maastrichtian	*Aquilapollenites quadrilobus*	Latest Maastrichtian
					*Kurtzipites circularis/simplex*	Late Maastrichtian	*Tricolpites (Gunnera) microreticulatus*	Late Maastrichtian
					*Ulmoideipites tricostatus*	Late Maastrichtian		
					*Ghoshispora bella*	Late Maastrichtian		
					*Triplanosporites sinuosus*	Late Maastrichtian		
P15 (61622−1)	MB	79	Latest Maastrichtian	high	*Triplanosporites sinuosus*	Late Maastrichtian	*Aquilapollenites quadrilobus*	Latest Maastrichtian
					*Pandaniidites typicus*	Latest Maastrichtian	*Aquilapollenites reticulatus*	Latest Maastrichtian
					*Styxpollenites calamitas*	Late Maastrichtian	*Leptopecopites pocockii*	Late Maastrichtian
					*Wodehouseia spinata*	Latest Maastrichtian	*Liliacidites complexus*	Latest Maastrichtian
							*Styxpollenites calamitas*	Latest Maastrichtian
							*Tricolpites (Gunnera) microreticulatus*	Late Maastrichtian
P16 (61722−4)	MB	89	Late Maastrichtian	high	*Sparganium* sp	Late Maastrichtian	*Aquilapollenites quadrilobus*	Latest Maastrichtian
					*Ulmipollenites krempii* 4-pore	Late Maastrichtian	*Tricolpites (Gunnera) microreticulatus*	Late Maastrichtian
					*Ulmipollenites krempii* 3-pore	Late Maastrichtian		
P17 (61722−6)	MB	90.3	mid-late Maastrichtian	Low-moderate	*Tricolpites (Gunnera) microreticulatus*	Mid-Maastrichtian	*Tricolpites (Gunnera) microreticulatus*	Late Maastrichtian

In spite of the various reworked material, all palynology samples from the Dueling Section and the Upper McGinnis Butte Section yielded late to latest Maastrichtian age estimates (Table 1), based on age ranges for palynomorphs reported from North America, with special emphasis placed on age ranges for palynomorphs reported from the Western Interior Seaway and the palynostratigraphy of Montana and surrounding areas more specifically [81–88]. No diagnostic Paleogene taxa were encountered in the highest samples collected ~10 m below the top of the MB section. However, the distinctive biostratigraphic marker species *Aquilapollenites collaris* was observed to be common and first appears 40 m above the base of the DD/MB section or ~9–10 m above the “Dueling Dinosaurs” level. *A. collaris* is restricted to the middle and upper portions of the Hell Creek Formation and part of Subzone C of the *Wodehouseia spinata* Assemblage Zone [82]. The lack of this taxon in the lower 40 m of the section is consistent with this interval correlating to the lower portion of the Hell Creek Formation (Fig 3). Kaskes et al. [37] in their stratigraphic investigation of the Naturalis *T. rex* locality, which is also located on the Murray Ranch ~8 km to the east of the DD section, Kaskes et al. [37] noted that the site was within C30N, but below the first appearance of *A. collaris,* suggesting an age between c.66.7–67.2 for that specimen. Interestingly, the first appearance of *A. collaris* in the DD/MB sections is also ~10 m above the “Dueling Dinosaurs” fossil level, suggesting that the “Dueling Dinosaurs” and the Naturalis *T. rex* are likely both located at a similar stratigraphic level within the lower portion of the Hell Creek Formation [37]. Kaskes et al. [37] placed the Naturalis site within the informal member nomenclature of Hartman et al. [2014], correlating the site with the top of the informal lower member. We avoid using this informal nomenclature as the sandstone beds used to define these boundaries cannot be confidently identified in the study area.

Along with palynology, kerogen analysis of the organic residue was also conducted on all samples. Charcoal is an abundant component of the kerogen in several samples, suggesting that forest fires were not uncommon in the area. Based on the thermal alteration index determined from pollen, the Hell Creek Formation has undergone minor burial, with samples yielding estimated Ro% (vitrinite reflectance) values between 0.25 and 0.34.

### Magnetostratigraphy

**Fig 5:**
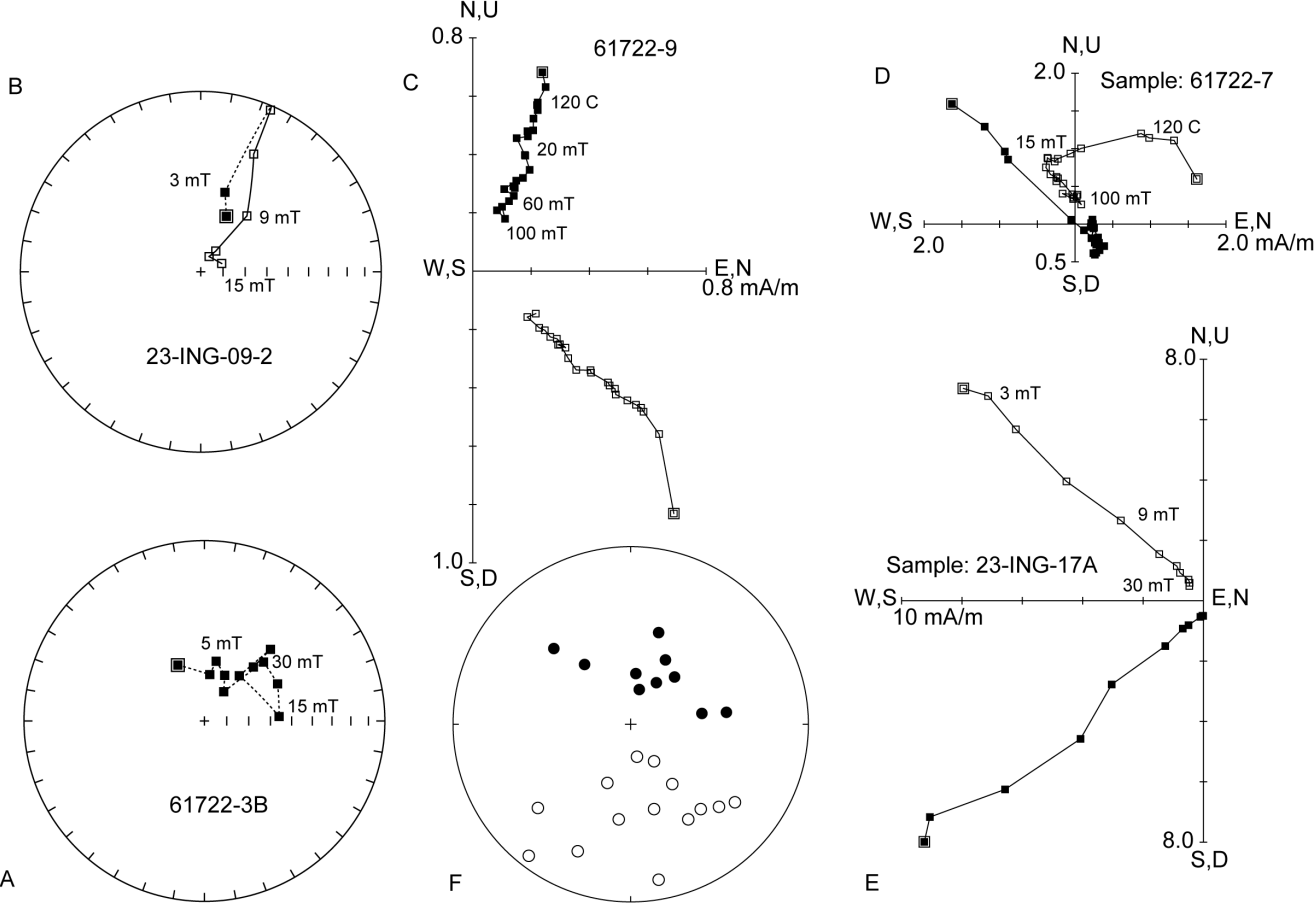
Paleomagnetic data. (A) Equal area projection of demagnetization data for a normal polarity sample illustrating clustering of remanence directions characterized by a Fisher mean [60]. (B) Equal area projection of demagnetization data for a reversed polarity sample where the remanence directions follow a great circle trajectory. (C-E) Vector endpoint diagrams for selected samples. Vector endpoints are labeled with alternating field intensity (mT) or temperature (°C). 61722−9 (normal polarity), 61722−7 (reversed polarity), and 23-ING-17A (reversed polarity) show linear decay toward the origin and are characterized by PCA. (F) Equal area projection of sample characteristic remanence directions for samples characterized by PCA or Fisher mean used for polarity interpretations.

Most paleomagnetic samples exhibited stable demagnetization with relatively clear polarity determinations (Fig 5). Although the paleomagnetic samples and associated sites included in the magnetostratigraphic analysis separated themselves into corresponding reverse or normal polarities (Fig 5F), they did not pass the reversal test, likely due to the relatively small sample size and/or incomplete removal of a weathering-induced present-day normal overprint. All magnetostratigraphic samples collected from the DD section yielded normal polarity consistent with deposition during the C30n chron (Table 2). In contrast, a clear polarity reversal was documented between sites M8 and M9 within the MB section (Table 2; Fig 3). Approximately 5 meters of stratigraphy separates these two sites. Included in this interval is an erosional surface at the base of the ~ 10 m thick sandstone that caps the section (Figs 2 and 3). We infer that the C30n/C29r reversal occurs at this erosional contact, which is at the ~ 91.8 m level in the MB section (Fig 3).

**Table 2:** Summary of Paleomagnetic Data.

Field Sample #	Site #	Strat. Section	Strat. Level (m)	GPS Lat/Long (WGS 84)	NRM intensity (A/m)	Total Steps	Dec. (deg)	Inc. (deg)	MAD/a95	Steps in Calc	PCA/M/GC	VGP Lat (Site)	Polarity (Site)
61822−2	M1	DD	33.2	46.941829°/ −107.353089°	2.07E-03	23	322.2	55	5.3	22	PCA	59.7	N
61822−7	M2	DD	48	46.942378°/ −107.350125°	2.74E-04	17	13.5	73.8	18.5	11	PCA	74.9	N
23-DD-505B	M3	DD	53.7	46.941178°/-107.350417°	1.90E-03	16	81.1	56.7	8.5	6	M		
23-DD-505C-1	M3	DD	53.7	46.941178°/-107.350417°	3.43E-03	8	31.8	67.5	12.6	7	PCA		
23-DD-505C-2	M3	DD	53.7	46.941178°/-107.350417°	2.48E-03	18	28.1	56	9.7	12	PCA	56.6	N
61822−9	M4	DD	56.1	46.941178°/-107.350417°	3.71E-04	23	5.9	66.8	10.3	22	PCA	85.4	N
61822−11	M5	DD	60.8	46.940924°/-107.349887°	4.04E-04	24	314.3	39	11.4	22	PCA	45.7	N
61722−9	M6	MB	55.4	46.944720°/-107.381756°	1.10E-03	23	16.7	45.2	10.4	23	PCA	65.9	N
61722−2	M7	MB	75.8	46.944615°/ −107.382115°	6.79E-04	18	82.8	44.8	21.6	4	M	23.7	N
61722-3B	M8	MB	88	46.945010°/ −107.382430°	7.22E-05	12	43	60.1	8.4	11	M	58.9	N
23-ING-17A	M9	MB	93.4	46.945053°/ −107.382539°	1.39E-02	21	227.8	−30.2	2.8	11	PCA		
23-ING-17B	M9	MB	93.4	46.945053°/ −107.382539°	1.24E-02	27	170	−12	1.8	11	PCA		
23-ING-17C	M9	MB	93.4	46.945053°/ −107.382539°	2.45E-02	15	202.7	−23.3	2.1	12	PCA	−51.9	R
23-ING-16A	M10	MB	95	46.945053°/ −107.382539°	4.60E-03	13	148.1	−69.8	9.7	9	M		
23-ING-16B	M10	MB	95	46.945053°/ −107.382539°	3.54E-03	25	201.5	−60.8	9.8	21	M	−86.4	R
23-ING-301-B	M11	MB	95.4	46.945053°/ −107.382539°	9.64E-04	4	149	−37.4	2.8	4	M		
23-ING-301C	M11	MB	95.4	46.945053°/ −107.382539°	4.51E-02	18	133.2	−32.2	16.2	4	M	−47.9	R
23-ING-02	M12	MB	96.4	46.945053°/ −107.382539°	1.90E-03	17	145.6	−56.4	8	4	M		
23-ING-09–1	M12	MB	96.4	46.945053°/ −107.382539°	1.85E-03	9	187	−45.2	14.6	3	M		
23-ING-09–2	M12	MB	96.4	46.945053°/ −107.382539°	1.39E-03	12	205.2	85.8	6.6	12	GC	−73.9	R
23-ING-13	M13	MB	96.7	46.945053°/ −107.382539°	3.96E-03	13	127	−27.8	4.3	9	PCA		
61722−7	M13	MB	96.7	46.945053°/ −107.382539°	2.38E-03	25	165	−48.9	14.2	14	PCA	−51.7	R
1022-1A	M14	MB	101.7	46.945123°/ −107.382492°	2.61E-03	21	140.5	−37.9	22	5	PCA		
1022-1B	M14	MB	101.7	46.945123°/ −107.382492°	1.43E-03	13	221.3	82.8	14.2	10	GC		
1022−2	M14	MB	101.7	46.945123°/ −107.382492°	3.48E-02	15	169.1	−74.8	4.4	9	PCA		
1022−4	M14	MB	101.7	46.945123°/ −107.382492°	5.57E-02	16	217.7	−7.1	10.2	9	PCA	−70.3	R

Total Steps – total number of demagnetization steps.

Dec/Inc for PCA line, Mean, or GC strike/dip of plane.

MAD/a95 - MAD for PCA lines and GC, a95 for Means.

Steps in Calc – Number of steps included in PCA, Mean, or GC calculation.

PCA/M/GC - Sample characterized by Principle Component Analysis (PCA), Mean (M), or Great Circle (GC).

VGP Lat – Latitude of Virtual Geomagnetic Pole for site.

Polarity – Site polarity determination based on site VGP latitude.

### Bentonite identification and sampling

Field identification and sampling of the two bentonites that we interpret as primary volcanic ash fall deposits and utilized for U-Pb dating followed the approaches outlined in previous studies [30–34]. In this study, field identification involved identifying resistant 20–30 cm-thick claystone beds that typically stand out on the landscape because of their distinctive ‘popcorn’ appearance (Fig 2A), which is caused by the swelling nature of pure smectite clays that form from weathering of the volcanic ash [30,31,35–37]. This was followed by excavating the beds ~0.5–2 m into the hill to expose fresh, unweathered rock (Fig 2B). Once exposed, careful visual evaluation was conducted to provisionally determine if the bentonites are pure ash-fall tuffs or reworked tuffaceous (volcaniclastic) sediments. Reworked tuffaceous beds are typically silty or sandy bentonite, whereas unreworked air fall ashes are characterized by a pure smectite clay layer, which is typically only a few centimeters to decimeters thick (30–31, 90–91). These thin beds were recognized by their pistachio green color and waxy luster (indicative of pure claystone) with abundant, floating grains of euhedral black biotite crystals that can be seen with a hand lens. In the study area, of the roughly 5–6 prospective bentonites that were initially identified, most proved not to match the criteria for a pure ash fall tuff upon excavation. However, both the Dueling and Ingomar bentonites passed these tests and stood out for their prolific ‘popcorn’ texture. They are also readily traceable across much of the study area, unlike the reworked tuffaceous beds.

Once these field tests were conducted, further examinations were made to firmly establish the nature of the Dueling and Ingomar bentonite beds as primary ash fall deposits. To do this, ~ 4 kg of fresh bentonite was collected from both of the excavated beds, followed by clay separation to concentrate the phenocrysts and/or detrital clasts, following the steps outlined in Hoke et al. (2014). The silt/sand-sized fractions of each sample were further separated into heavy and light mineral fractions on a Wilfley Table and with high-density liquids. In both cases, the resultant light fractions lacked evidence of rounded detrital quartz, feldspar, or lithic grains that would indicate reworking. The presence of euhedral sanidine (and other feldspars) and bi-pyramidal quartz grains in the light fraction of each sample, along with a significant population of fresh, euhedral biotite, apatite, titanite, and zircon phenocrysts in the heavy mineral fraction was verified (Fig 2E and F). In addition, many of the zircon crystals are elongated and exhibit large glass (melt) inclusions oriented parallel to their long axis, which provides evidence against substantial sediment transportation and reworking (30–34). Thin sections of both bentonites reveal pure smectite clays with isolated, floating euhedral mineral phenocrysts consistent with the derivation of the bentonite from altered volcanic ash (glass); however, as expected, the volcanic glass (ash) in both samples is entirely altered to smectite clay, and no relic glass shards were visible in the thin sections (30–34, 95–97).

### CA-ID-TIMS U-Pb geochronology and Bayesian model results

**Fig 6:**
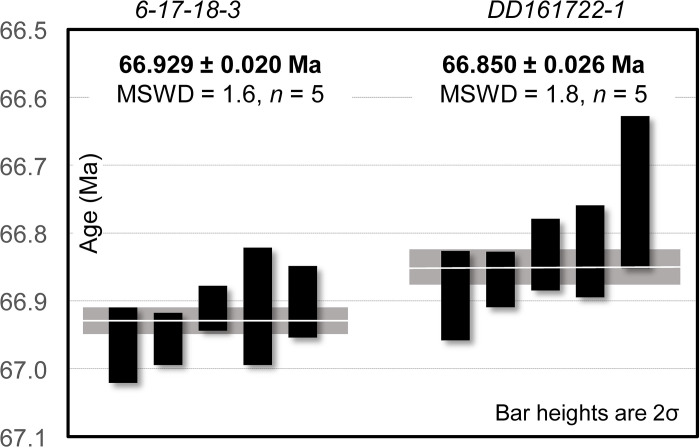
U-Pb CA-ID-TIMS ranked age plots for analyzed zircons from the Dueling Bentonite and the Ingomar Bentonite.

Two new U-Pb CA-ID-TIMS ages for the Hell Creek Formation from bentonite beds were successfully obtained from the study area. The Dueling Bentonite yielded a weighted mean ^206^Pb/^238^U age of 66.929 ± 0.020/0.021/0.076 Ma with a mean square weighted deviation (MSWD) of 1.6 based on five statistically coherent zircon analyses (Fig 6; Table 3). The stratigraphically higher Ingomar Bentonite (4.9 m above the Dueling Bentonite bed) yielded a slightly younger weighted mean age of 66.850 ± 0.026/0.041/0.082 Ma with a MSWD of 1.8 based on 5 zircon analyses (Fig 6; Table 3). We interpret the new U-Pb dates as the best approximations of zircon crystallization age and thus the age of deposition of the corresponding bentonites. Our interpretation is supported by petrographic evidence and zircon morphologies (Fig 2), as well as lack of dispersion in U-Pb analyses and bentonite age conformity to stratigraphic superposition. Both new ages, the recalculated age of the C30n/C29r boundary (66.48 ± 0.55 Ma) and associated stratigraphic positions were utilized to construct the Bayesian age-stratigraphic model shown in Fig 3. Unfortunately, the large uncertainty on the recalculated C30n/C29r chron boundary age and the unconstrained top of the section above this boundary (i.e., no K/Pg boundary present) means that the age model has limited utility for constraining the top of the section (see discussion). However, because the “Dueling Dinosaurs” level is tightly bracketed between the two dated bentonites, a precise model age of 66.897 + 0.023/-0.028 Ma (asymmetric uncertainty) for the fossil bed was established (Fig 3). Complete U-Pb analytical data are presented in Table 3.

**Table 3:** U-Pb isotopic data.

					Ratios								Ages (Ma)						
Sample	Pb(c)	Pb*	U	Th	206 Pb	208 Pb	206 Pb		207 Pb		207 Pb		206 Pb		207 Pb		207 Pb		corr.
Fractions	(pg)	Pb(c)	(pg)	U	204 Pb	206 Pb	238 U	err	235 U	err	206 Pb	err	238 U	err	235 U	err	206 Pb	err	coef.
(a)	(b)		(c)		(d)	(e)	(f)	(2σ%)	(f)	(2σ%)	(f)	(2σ%)		(2σ)		(2σ)		(2σ)	
*Sample 6-17-18-3 (Dueling Bentonite)*																			
**z1**	0.48	22.3	1008	0.42	1372.2	0.135	0.010442	(.08)	0.06861	(.94)	0.04768	(.91)	**66.965**	**0.056**	67.38	0.61	82	22	0.32
**z4**	0.33	36.2	1096	0.47	2192.1	0.149	0.010441	(.06)	0.06844	(.60)	0.04756	(.58)	**66.956**	**0.039**	67.22	0.39	77	14	0.34
**z2**	0.54	44.0	2218	0.49	2646.9	0.155	0.010434	(.05)	0.06823	(.49)	0.04745	(.47)	**66.911**	**0.033**	67.02	0.32	71	11	0.28
**z5**	0.55	12.5	649	0.44	777.0	0.140	0.010433	(.13)	0.06876	(1.65)	0.04782	(1.61)	**66.908**	**0.087**	67.5	1.1	89	38	0.32
**z3**	0.42	21.8	873	0.43	1343.8	0.136	0.010432	(.08)	0.06850	(.95)	0.04765	(.93)	**66.901**	**0.053**	67.28	0.62	81	22	0.32
*Sample DD161722−1 (Ingomar Bentonite)*																			
**z3**	0.29	20.3	541	0.50	1230.0	0.159	0.010431	(.10)	0.06855	(1.17)	0.04769	(1.15)	**66.892**	**0.066**	67.32	0.76	83	27	0.24
**z2**	0.26	55.0	1337	0.47	3312.9	0.152	0.010427	(.06)	0.06851	(.60)	0.04767	(.58)	**66.868**	**0.041**	67.28	0.39	82	14	0.42
**z4**	0.33	32.0	982	0.43	1959.2	0.139	0.010421	(.08)	0.06870	(.70)	0.04783	(.69)	**66.832**	**0.053**	67.47	0.46	90	16	0.24
**z1**	0.32	20.3	583	0.66	1175.3	0.210	0.010420	(.10)	0.06843	(2.71)	0.04765	(2.69)	**66.827**	**0.068**	67.2	1.8	81	64	0.17
**z5**	0.34	13.2	412	0.48	806.6	0.154	0.010407	(.17)	0.06896	(2.53)	0.04808	(2.49)	**66.74**	**0.11**	67.7	1.7	102	59	0.27

**Notes:**

(a) Thermally annealed and pre-treated single zircon. Data used in age calculation are in bold.

(b) Total common-Pb in analyses.

(c) Total sample U content.

(d) Measured ratio corrected for spike and fractionation only.

(e) Radiogenic Pb ratio.

(f) Corrected for fractionation, spike and blank. Also corrected for initial Th/U disequilibrium using radiogenic ^208^Pb and Th/U_magma_ = 2.8.

All common Pb assumed to be laboratory blank. Total procedural blank less than 0.1 pg for U.

Blank isotopic composition: ^206^Pb/^204^Pb = 18.15 ± 0.47, ^207^Pb/^204^Pb = 15.30 ± 0.30, ^208^Pb/^204^Pb = 37.11 ± 0.87.

Corr. coef. = correlation coefficient.

Ages calculated using the decay constants λ_238_ = 1.55125E-10 y^-1^ and λ_235_ = 9.8485E-10 y^-1^ (Jaffey et al. 1971).

## Discussion

### Stratigraphy and age of the Dueling Dinosaur and McGinnis Butte sections

To date, the Dueling and Ingomar bentonites are the oldest dated tephra beds in the Hell Creek Formation. The Null Coal tephra (^40^Ar/^39^Ar age of 66.289 ± 0.051 Ma; [20]) is located several meters above the C30n/C29r chron boundary in the type area, whereas the Dueling and Ingomar bentonites occur ~65–70 m below this chron boundary. In addition, these two bentonites occur 7 and 12 m below the first appearance of pollen *A. collaris*, which is restricted to the informal middle member of the Hell Creek Formation elsewhere [83,89]. Thus, the report herein of the Dueling and Ingomar bentonite ages and their stratigraphic position significantly expand the range of datable tephra deposits in the Hell Creek Formation while also demonstrating the potential for better age control throughout the formation.

Results of our Bayesian age-stratigraphic model indicate an age of 66.897 + 0.023/-0.028 Ma for the “Dueling Dinosaurs” fossils and based on comparison of the pollen and stratigraphy of Kaskes et al. [37] for the Naturalis *T. rex* site (~8 km away), we conclude that both specimens are roughly the same age. Due to a lack of continuous exposure between the two sites, however, we cannot demonstrate whether both specimens occur within the same or different channelized sandstone complexes. Ongoing sedimentological investigation of channel sandstones in the study area suggests they have an anabranching morphology with multiple active channels at the same level (particularly in the lower half of the formation). For this reason and the likelihood that any aerially widespread channelized sandstone bodies are time-transgressive, we challenge the assumption that channelized sandstones provide a viable basis for lithostratigraphic or chronostratigraphic correlation in the Hell Creek Formation (e.g., JenRex Sandstone, Apex Sandstone, etc. [11,26]). Our observations while walking out the Dueling bentonite in the study area reveal it to be highly discontinuous, primarily due to the anastomosed fluvial morphology in which multiple channels occur at the same stratigraphic level and separate broad packages of floodbasin mudrock interpreted as interfluves (Fig 3; see also [90]).

### Age of base of Hell Creek Formation and its duration

The age-stratigraphic model produced in this study predicts an approximate age of 67.102 + 0.710/-0.173 Ma for the base of the DD/MB composite section, which we propose as the base of the Hell Creek Formation in the study area. This model age has the advantage of being generated using the first dated ash beds from the lower portion of the formation, although we consider this a minimum age for the base of the Hell Creek Formation, regionally, since we cannot confidently confirm that the base of the section sits at the Fox Hills Formation contact in the study area. Furthermore, the precision of the basal model age is poor due to a sparsity of dated horizons close to the formation contact, whereby variation in sediment accumulation rates below the Dueling bentonite could result in a younger or, especially, older true age for the contact. Comparison to other published estimates for the basal age of the Hell Creek Formation supports the age calculated here, which is essentially the same as the minimum age of 67.2 Ma suggested by Eberth and Kamo [25] based on their correlations with the Battle Formation in Alberta.

The C30n/C29r chron boundary age was crucial in generating the age-stratigraphic model herein, although determining the ideal age and uncertainty to use for this boundary was challenging. Sprain et al. [21] reported an age of 66.311 ± 0.102 Ma (2σ analytical uncertainty) using linear extrapolation from ^40^Ar/^39^Ar geochronology of volcanic ash horizons close to the chron boundary in the Hell Creek Formation type area near Fort Peck Reservoir. Application of this age for our purposes would require the addition of systematic errors, including decay constant uncertainty for both the boundary age and our dated bentonites, which would have a significant impact on the apparent precision of the model age for the Dueling fossil locality. Alternatively, Clyde et al. [27] calculated an age of 66.436 ± 0.039 Ma (2σ analytical uncertainty) for the chron boundary as it occurs in the Denver Basin based on high-precision U-Pb zircon ages from volcanic ash horizons in the Kiowa core. This age is statistically indistinguishable from that of Sprain et al. [21] even before considering systematic error, although linear interpolation used by Clyde et al. [27] was applied across a much larger stratigraphic interval of ~20 m with no additional age constraint below the chron boundary. As a result, the propagated uncertainty does not account for variability in sediment accumulation rates and is thus likely to significantly overestimate precision. To ensure the C30n/C29r chron boundary age was compatible with our DD/MB model, we recalculated the Clyde et al. [27] U-Pb zircon-derived age using a Bayesian age-stratigraphic model for the Kiowa core. Our recalculated chron boundary model age of 66.48 + 0.55/-0.17 Ma more appropriately represents stratigraphically propagated uncertainty but appears significantly less precise owing to a lack of dated horizons low in the Kiowa core. This issue is also present in the DD/MB model, raising concerns over the utility of both the recalculated chron age and the basal DD/MB model age. Future efforts to date volcanic ash horizons close to and below the C30n/C29r chron boundary would greatly improve temporal resolution of the Hell Creek Formation, as would a similar sampling approach above and below the basal contact of the formation.

Although the K-Pg boundary was not identified in our study area, we can estimate a total duration for the Hell Creek Formation in the study area to represent ~1.08 Myr, based on our model age for the base of the DD/MB section (67.102 + 0.710/-0.173) and the Clyde et al. [27] K-Pg boundary age of 66.021 ± 0.024 Ma (2σ analytical uncertainty), which is within error of the K-Pg age of Sprain et al. [21] and the GTS2020 [79]. The duration of deposition represented by the 109-meter-thick DD/MB composite section (missing upper contact) is approximately 0.737 Myrs, equating to an average sediment accumulation rate of 25.8 cm/kyr across the 109-metre-thick section. Based on the time interval between top of the DD/MB section (model age of 66.365 + 0.406/-0.713 Ma) and the Clyde et al. [27] K/Pg age of 66.02 Ma combined with the average sediment accumulation rate, the model predicts that ~90 m of Hell Creek Formation strata may be missing above the MB section for a total formation thickness of ~200 m in the study area at the time of deposition, not accounting for possible variation in sediment accumulation rates or unconformities. This would make the section considerably thicker than what has been reported in the type section and most other locations. Based on observations of Fort Union Formation cropping out several kms north of the study area, we suspect that 200 m is too thick an estimate. However, the thickest sections of the Hell Creek Formation/equivalent strata do reportedly thicken significantly to the south, even though minimal west-east thickening has been observed [91].

Using a previous K/Pg boundary age of 65.51 Ma, Hicks et al. [22] estimated the duration of the Hell Creek Formation at 1.36 Myr; however, using the currently accepted boundary age of around 66.021 Ma [21], their estimate would be closer to a duration of between ~700–850 kyr in western North Dakota. This is somewhat shorter in duration than what we report here, and may be explained by a possible eastward thinning, time-transgressive nature of the Hell Creek clastic wedge. It should be expected that the temporal duration of the Hell Creek Formation should increase to the west, and currently, the DD/MB Hell Creek Formation section is among the furthest west stratigraphic sections published for the formation, located south and slightly west of the type section at Flagg Butte near Reid Creek [11]. Although the stratigraphic relationships of the upper Hell Creek Formation are well resolved in the type area and well-studied sections in North Dakota, much work remains to understand the regional stratigraphic relationships of the Hell Creek Formation and correlative units, such as the Lance and St. Mary River formations, outside of these areas. Furthermore, until regional (i.e., basin wide) documentation of Hell Creek stratigraphic relationships is better resolved, fundamental uncertainties regarding the nature and timing of dinosaur (and other vertebrate) coevolution with their environment remain.

### Source of ash and correlation of bentonites within the Hell Creek Formation

Unlike most previous studies where tephras have primarily been identified within lignite seams in the Hell Creek and Fort Union Formation, we report two new thin, but pure bentonite beds from the lower portion of the Hell Creek Formation. These are the first two dated tephras documented from anywhere in the formation below the null coal ash (uppermost Hell Creek Formation). Their relative thinness and discontinuous but laterally reoccurring distribution, coupled with relatively fine phenocryst sizes (< 500 µm), suggest that the ashes are reasonably far traveled, or alternatively from less explosive, localized eruptions potentially emanating from nearby alkaline volcanic centers in the Central Montana Alkalic Belt. Campanian bentonites across the Western Interior Basin (WIB), from Alberta, Montana, Wyoming, Nebraska, and the Dakotas, have been linked to the Elkhorn Mountain Volcanics [92–96]. However, the source of Maastrichtian ashes within the WIB remains far more speculative, with limited previous work.

As an example, the ages of the Dueling Bentonite and the Battle Bentonite in Alberta are within error of each other [25], leading us to speculate that both bentonites are part of the same ashfall event that may represent a regional marker horizon with a minimum area of 650 km^2^. Confirmation requires testing through geochemical fingerprinting, but it poses the question of just how extensive these ash beds are across western North America and whether they represent locally derived ashfall/ashflow deposits or large, regional tephra blankets from a single source. Given that so little is known about the nature and source of Maastrichtian (and Danian) bentonites in the Western Interior Basin, this presents a fertile area for future investigation. It is highly likely that with further exploration of the lower to middle portion of the Hell Creek Formation, additional ash beds will be discovered that will assist with this provenance interpretation, as well as lead to even better age control in the formation and establishment of regional marker horizons for correlation.

## Conclusions

Given the scientifically significant nature of the ‘Dueling Dinosaurs” fossil locality, collected over two decades ago and recently accessioned into the North Carolina Museum of Natural Sciences collections, the primary aim of this study was to return to the Murray Ranch to establish a detailed stratigraphic context for the “Dueling Dinosaurs” (NCSM 40000 and NCSM 40001). Due to the remote and poorly understood stratigraphy of the Hell Creek Formation in the extensive exposures in central Montana to the south and west of Jordan Montana, it was necessary to establish a composite section for the Murray Ranch and nearby McGinnis Butte by utilizing a variety of dating approaches to date and correlate the stratigraphy and significant vertebrate fossils, including NCSM 40000 and NCSM 40001. Lithostratigraphy, coupled with palynology and magnetostratigraphy, demonstrates that the composite section in the region is ~ 109 m thick, beginning at or close to the Fox Hills Formation, and continuing to close to the top of the Hell Creek Formation, but ending just prior to the K-Pg boundary in lower C29R. Identification and high-precision CA-ID-TIMS U-Pb zircon dating of two bentonite beds bracketing the fossil locality are the stratigraphically lowest ever reported from the Hell Creek Formation. Based on these geochronologic results and Bayesian age-stratigraphic modelling, the “Dueling Dinosaurs” (NCSM 40000 and NCSM 4000) locality is assigned a precise model age of 66.895 + 0.023/-0.027 Ma, which roughly correlates with the top of the informal lower member. The two new U-Pb ages were also used to model a minimum age of 67.102 + 0.710/-0.173 Ma for the base of the Hell Creek Formation in the study area. This date is consistent with the age proposed for the base of the formation by Eberth and Kamo [25] and addresses the significant amount of uncertainty regarding the age of the base and total duration of the formation. Indeed, the CA-ID-TIMS U-Pb age of the Battle Bentonite (66.936 ± 0.047 Ma) utilized by Eberth and Kamo [25] to make these estimates is within analytical uncertainty of the age of the Dueling Bentonite (66.929 ± 0.020 Ma). It is thus likely that the Dueling/Battle bentonites represent the same ash bed forming a regional marker horizon with a minimum total areal extent of 650 km^2^. This presents a much-needed opportunity for correlation of the lower/mid Hell Creek Formation and equivalent strata, flora, and fauna across the Western Interior Basin in the US and Canada. It is also important to point out that there are many such specimens collected by private collectors that have ended up in museums, and that these specimens could also greatly benefit from follow-up geological investigations that have the potential to add important context to these specimens and can be achieved long after the original excavations took place.

## Supporting information

S1 File BChron Tables S1-S6. S1 Table. DD Bchron Input Data. S2 Table DD Bchron R Script. S3 Table DD Bchron Model Ages. S4 Table Kiowa Bchron Input Data. S5 Table Kiowa Bchron R Script. S6 Table Kiowa Bchron Model Ages.
(XLSX)
